# Downregulation of an Evolutionary Young miR-1290 in an iPSC-Derived Neural Stem Cell Model of Autism Spectrum Disorder

**DOI:** 10.1155/2019/8710180

**Published:** 2019-05-02

**Authors:** Dalia Moore, Brittney M. Meays, Lepakshe S. V. Madduri, Farah Shahjin, Subhash Chand, Meng Niu, Abrar Albahrani, Chittibabu Guda, Gurudutt Pendyala, Howard S. Fox, Sowmya V. Yelamanchili

**Affiliations:** ^1^Department of Pharmacology and Experimental Neuroscience, University of Nebraska Medical Center, Omaha, NE, USA; ^2^University of Arkansas for Medical Sciences, Little Rock, AR, USA; ^3^Department of Genetics Cell Biology and Anatomy, University of Nebraska Medical Center, Omaha, NE, USA; ^4^Department of Anesthesiology, University of Nebraska Medical Center, Omaha, NE, USA

## Abstract

The identification of several evolutionary young miRNAs, which arose in primates, raised several possibilities for the role of such miRNAs in human-specific disease processes. We previously have identified an evolutionary young miRNA, miR-1290, to be essential in neural stem cell proliferation and neuronal differentiation. Here, we show that miR-1290 is significantly downregulated during neuronal differentiation in reprogrammed induced pluripotent stem cell- (iPSC-) derived neurons obtained from idiopathic autism spectrum disorder (ASD) patients. Further, we identified that miR-1290 is actively released into extracellular vesicles. Supplementing ASD patient-derived neural stem cells (NSCs) with conditioned media from differentiated control-NSCs spiked with “artificial EVs” containing synthetic miR-1290 oligonucleotides significantly rescued differentiation deficits in ASD cell lines. Based on our earlier published study and the observations from the data presented here, we conclude that miR-1290 regulation could play a critical role during neuronal differentiation in early brain development.

## 1. Introduction

During brain development, the regulation of cellular proliferation, which ensures the renewal of neural progenitor cells as well as the process of neural fate determination, results from highly complex processes. These processes are in turn fine-tuned by several regulatory networks to function in synchrony. Keys among these regulatory pathways are critical epigenetic factors such as microRNAs (miRNAs) [1]. Moreover, a recent discovery of several evolutionary young miRNAs not conserved beyond primates raised several possibilities for the role of such miRNAs in human-specific disease processes [2, 3]. It is still speculative if these higher species-specific miRNA pools are responsible for shaping higher order functions such as in the human brain. A previous work from our group has identified one such miRNA, miR-1290, found only in great apes (gorillas, chimpanzees, and humans), to be essential in neural stem cell (NSC) proliferation and neuronal differentiation events [4]. We have shown that miR-1290 targets crucial cell cycle genes and acts as an upstream regulator during neuronal differentiation. Several recent studies using *in vitro* models of neuronal development have identified that neuronal differentiation and cell cycle processes were altered significantly in idiopathic autism spectrum disorder (ASD) [5–7]. A recent study in autistic toddlers has also shown significant alterations in cell cycle networks [8].

Here, we examined the role of miR-1290 in ASD, by utilizing induced pluripotent stem cell- (IPSC-) derived neural stem cells (NSCs) from normal and idiopathic ASD individuals. We identified that miR-1290 is aberrantly expressed during neuronal differentiation in idiopathic ASD cell lines. Furthermore, we show that miR-1290 is secreted into the extracellular vesicles (EVs) during differentiation in ASD-NSCs. In conclusion, we show that mIR-1290 is an important factor for regulating neuronal differentiation during the early development of the human brain.

## 2. Materials and Methods

### 2.1. Materials

Neuronal stem cells (NSCs) were acquired from the National Human Neural Stem Cell Resource at the Children's Hospital of Orange Country (CHOC) Research Institute in Orange, California (http://nhnscr.org). IPSCs were derived from human dermal fibroblasts from control and clinically diagnosed male subjects. NSC cell lines used in the current study were obtained from single IPSC clones from the following patient IDs: unaffected controls (SC148 (Control #1), SC173 (Control #2), and SC176 (Control #3)) and idiopathic autism (SC101 (ASD#1), SC171 (ASD#2), and SC217 (ASD#3)) have been described previously [9]. All the normal and ASD patient IPSCs were karyotyped as mostly normal; however, it is to be noted there were a few clones that demonstrated one-cell nonclonal cell artifacts, which are mostly regarded as technical artifacts according to the karyotype reports (Supplementary File 1). NSCs were grown and maintained in growth media containing DMEM/F12 GlutaMax medium (Life Technologies, Lot: 10565-018), 10% Bit 9500 (Stem Cell Technologies, Cat. #: 09500), 10 *μ*M 0.2% heparin sodium salt in PBS (Stem Cell Technologies, Lot: 07980), 20 ng/mL recombinant human FGF basic (R&D, Lot: 234-FSE-025), and 20 ng/mL recombinant human EGF protein (R&D, Lot: 236-EG-200). Half medium exchanges were performed every other day, and vessels were passaged with Gentle Cell Dissociation Buffer (Stem Cell Technologies, 07174) when confluent. The use of human NSCs including their handling procedures was approved by the University of Nebraska Medical Center institutional review board (IRB # 616-15ES).

#### 2.1.1. Antibodies (Table 1)

### 2.2. Neuronal Differentiation

Glass coverslips were placed on 6-well plates and coated with Matrigel (Corning, 354230). Matrigel was kept on ice and was transferred to the 6-well plate with cold DMEM/F12 GlutaMax medium. Matrigel plates were incubated at room temperature for one hour and were then washed three times with DMEM/F12 GlutaMax medium. Growth media was added to the Matrigel plates. NSCs were plated at a density of 500,000 cells per well in a 6-well plate. After 1 day, the growth media was removed and replaced with neuronal differentiation medium. Neuronal differentiation medium consisted of Neurobasal medium (Life Technologies, 21103) supplemented with 1 : 100 GlutaMax (Life Technologies, 35050), 1 : 100 B27 (Life Technologies, 17504-044), 1 : 100 N_2_ (Life Technologies, 17502-048), 20 ng/mL BDNF (PeproTech, 450-02), 20 ng/mL GDNF (PeproTech, 450-10), 1 mM dibutyryl cAMP (Sigma-Aldrich, D0627), and 200 nM ascorbic acid. Half exchanges were done every other day to maintain neuronal growth. Differentiated neurons were taken down at different time points as described in Results.

### 2.3. Immunofluorescence

Cells were fixed in 4% paraformaldehyde made in 1x PHEM buffer (2x PHEM; 18.14 g Pipes, 6.5 g Hepes, 3.8 g EGTA, 0.99 g MgSO_4_, and pH 7.0 w/KOH) added to the coverslips for 20 minutes and then gently washed 3 times with PBS. Fixed cells were permeablized with 0.1% TritonX-100 in PBS for 15 minutes and then blocked in 10% normal goat serum (Vector Laboratories, S-1000) followed by primary antibody incubation overnight. Secondary Alexa-Fluor species-specific antibodies were used at 1 : 500 dilution with DAPI for nuclear staining (Thermo Fisher, 62248). Slides were mounted using Fluoromount-G (Southern Biotech, 0100-01).

### 2.4. Isolation of Extracellular Vesicles from Media

Serum-free media were removed from neurons that had differentiated from NSC. Protease inhibitors were added before spinning the samples at 20,000g for 20 minutes. The supernatant was filtered with a 0.22 *μ*m syringe filter. Using ultracentrifugation, samples were spun at 100,000g for 2 hours. Pelleted EVs were carefully resuspended in PBS and used immediately for further investigation.

### 2.5. Transmission Electron Microscopy (TEM)

For transmission electron microscopy (TEM), a 10 *μ*L drop of EV sample was placed on the grid (200-mesh copper grids coated with Formvar and silicon monoxide) and allowed to sit for 2 min. The excess solution was drawn off by filter paper, and the remaining thin film of sample was allowed to dry for 2 min. A drop of NanoVan negative stain was placed on the grid for 1 min. The excess negative stain was then drawn off by a filter paper and allowed to dry for at least 1 min before being placed in the TEM. Grids were examined on a Tecnai G2 Transmission Electron Microscope (built by FEI, Hillsboro, Oregon, USA) operated at 80 kV.

### 2.6. RNA Isolation Quantitative Real-Time PCR

RNA was isolated using Trizol (Life Technologies, 15596026). TaqMan mature miR assays for has-miR-1290 (Applied Biosystems, Carlsbad, CA, USA) were used to quantify according to the manufacturer's protocol. U6 snRNA was used as a housekeeping control. Reactions were performed, and calculations were made as described using the 2^ddCT^ method.

### 2.7. DOTAP Transfection

Cells were plated in growth media and on Matrigel-coated 24-well plates with 50,000 cells per well. After 1 day, cells were switched to differentiation media and transfected. MiR-1290 and miR-124 oligos were mixed with N-[1-(2,3-dioleoyloxy)propyl]-N,N,N-trimethylammonium methyl-sulfate (DOTAP) (Roche, Basel, Switzerland) and diluted separately in HBS buffer (20 mM HEPES, 150 mM NaCl, pH 7.4). The HBS containing either RNA or DOTAP was incubated for 5 minutes. Individual miRNAs were then added to the DOTAP and incubated for 20 minutes. A final volume of 50 *μ*L was added to each well of a 24-well plate, resulting in a final volume of 200 *μ*L. The ratio of DOTAP to miRNA used was 3 : 1.

### 2.8. Mimic Transfection

Synthetic mimics for mIR-1290 and miR-124 (Sigma-Aldrich, USA) were diluted and incubated in OptiMem for 5 minutes. These were then added to Lipofectamine RNAiMax reagent (Thermo Fisher, USA), incubated for 5 minutes, and then, 100 *μ*L of this mixture was added to each well of a 24-well plate. The final concentration of the miR mimic negative controls used was 1 *μ*M.

### 2.9. Coculture Experiments

ASD-NSCs at a density of 150,000 cells/well were plated on a Matrigel-coated 24-well plate containing coverslips. Corning Transwell inserts (Corning, NY) were also coated with Matrigel, and control cell lines were plated (50,000 cells/insert well). Insert pores (0.4 *μ*M) were large enough to allow the two conditioned media to shift but small enough to keep the cells and Matrigel separate. Both the inserts and wells stabilized in growth media for 24 h before switching to differentiation media. Coverslips were harvested at different time points and analyzed by immunostaining.

### 2.10. Western Blotting

Exosomal lysates were prepared using RIPA buffer (50 mM Tris/HCl, pH 8; 150 mM NaCl; 1% Nonidet P-40; 0.5% sodium deoxycholate; and 0.1% SDS), and protein quantification was carried out using Pierce BCA protein assay (Thermo Scientific, Rockford, IL, USA). Protein (5–15 *μ*g) was loaded in each lane of NuPAGE 4–12% Bis-Tris gels (Invitrogen). For EV proteins, gels were run under reducing conditions and separated proteins were transferred onto nitrocellulose membranes using iBlot (Invitrogen). The membranes were blocked in SuperBlock (TBS) blocking buffer (Thermo Scientific) and then incubated overnight at 4°C with primary antibody. This was followed by incubation with secondary antibody: HRP-conjugated anti-rabbit IgG (1 : 20,000; Thermo Scientific) or anti-mouse IgG (1 : 20,000; Thermo Scientific) for 1 h at room temperature. Blots were developed using SuperSignal West Pico Chemiluminescent Substrate (Thermo Scientific), imaged, and quantified using Carestream MI software (Carestream Health Inc., Rochester, NY, USA).

### 2.11. miRNA Northern Blotting

Northern blotting for miRNA detection was performed with minor modifications as described previously [4]. Briefly, 10 *μ*g of RNA denatured in Ambion gel loading buffer II (Life technologies) at 75°C for 15 min was loaded and ran on a 15% TBE-Urea gels (Invitrogen, Carlsbad, CA, USA) at 180 V for 1 h. The gel was then transferred onto a nylon membrane using an iBLOT DNA transfer stack (Invitrogen) as per the manufacturer's instructions. Subsequently, the membrane was crosslinked at 1200 kJ using a STRATALINKER (Stratagene, La Jolla, CA, USA). The membrane was then prehybridized in the prehyb buffer (Sigma-Aldrich) at 37°C for 1 h followed by the addition of 1.2 pmol/mL of LNA-modified 5′ and 3′ DIG-labeled hsa-miR-1290 probe (Exiqon) to the buffer and hybridization overnight at 37°C. The next day, the membrane was washed for 5 min with a low stringency wash buffer (2 × SSC, 0.1% SDS) followed by 2 × washes for 20 min each with a high stringency wash buffer (0.5 × SSC, 0.1% SDS) and a final wash for 20 min with ultrahigh stringency wash buffer (0.1 × SSC, 0.1% SDS). After the washes, the membrane was blocked for 1 h with 1 × blocking buffer for 1 h followed by incubation with anti-DIG AP antibody in a 1:  20 000 dilution in blocking buffer. Finally, DIG signal development was carried out using the DIG wash and block buffer set.

### 2.12. Bioinformatics

mir-1290 target genes were identified from multiple sources including miRTarBase, mirDB, IPA, and Targetscan to create a combined unique list of 4,644 genes. Of these, a reviewed set of 1,741 genes was further selected and mapped to a curated set of 603 ASD (http://asd.princeton.edu) genes to obtain the final set of 67 mir-1290-targeted ASD genes. These 67 genes were subjected to IPA and ClueGo analyses. ASD genes that affect different cellular processes such as behavior, embryonic development, nervous system development, and organismal development were mapped using the “Overlay” function in the IPA software.

### 2.13. Statistical Analysis

All experiments were performed in three individual patient samples from both the control and ASD groups (*n* = 3). Statistical analysis was performed using GraphPad Prism software (La Jolla, CA, USA). Student's *t*-test followed by the Holm-Sidak multiple comparison post-hoc test was performed, and the *P* value was calculated for each experiment. For all experiments with error bars, ±SEM was calculated to indicate the variation between experiments.

## 3. Results

### 3.1. Neuronal Proliferation, Differentiation, and miR-1290 Levels Are Altered in Idiopathic ASD-NSCs

**Figure 1:**
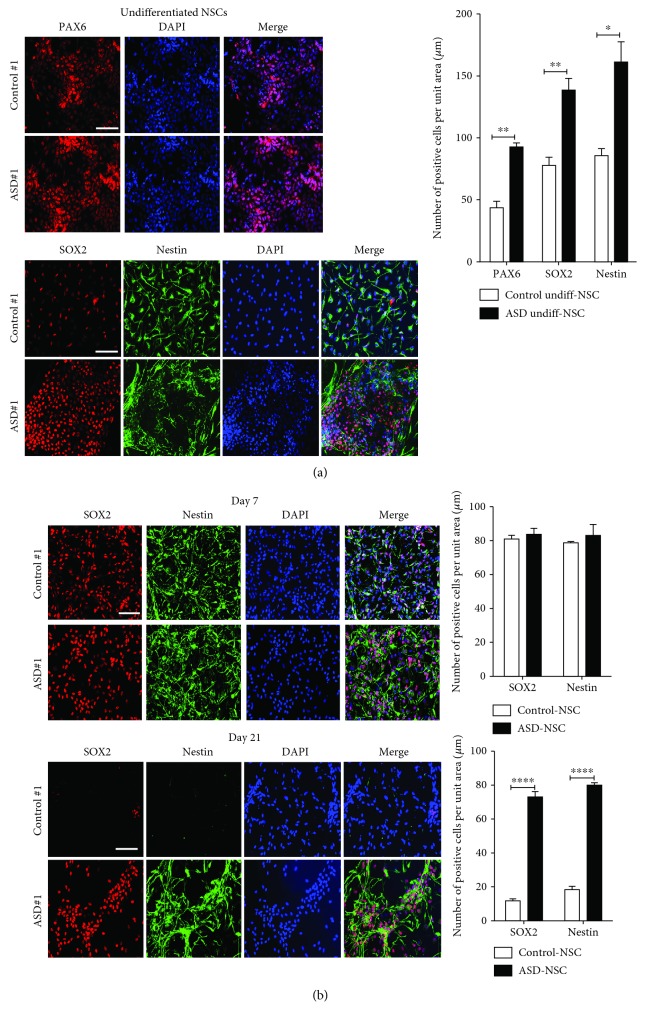
Neural progenitor markers are significantly enhanced in ASD-NSCs. (a) Immunostaining and statistical analysis indicate significantly more progenitor cells (Pax6^+^, Nestin^+^, and Sox2^+^) in ASD-NSCs than in control-NSCs. Bar = 20 *μ*m. *N* = 3, ^∗^ *P* < 0.01, and ^∗∗∗^ *P* < 0.001 (*n* = 3), determined by an unpaired *t*-test and corrected by the Holm-Sidak multiple correction post hoc test. (b) No differences in Nestin^+^ or Sox2^+^ NSCs were observed in day 7-differentiated control and ASD-NSCs; however, significantly more progenitor cells were observed in day 21-differentiated ASD-NSCs. ^∗∗∗∗^ *P* < 0.0001 (*n* = 3), determined by an unpaired *t*-test and corrected by the Holm-Sidak multiple correction post hoc test. Bar = 20 *μ*m. Data are represented as the mean ± SEM.

**Figure 2:**
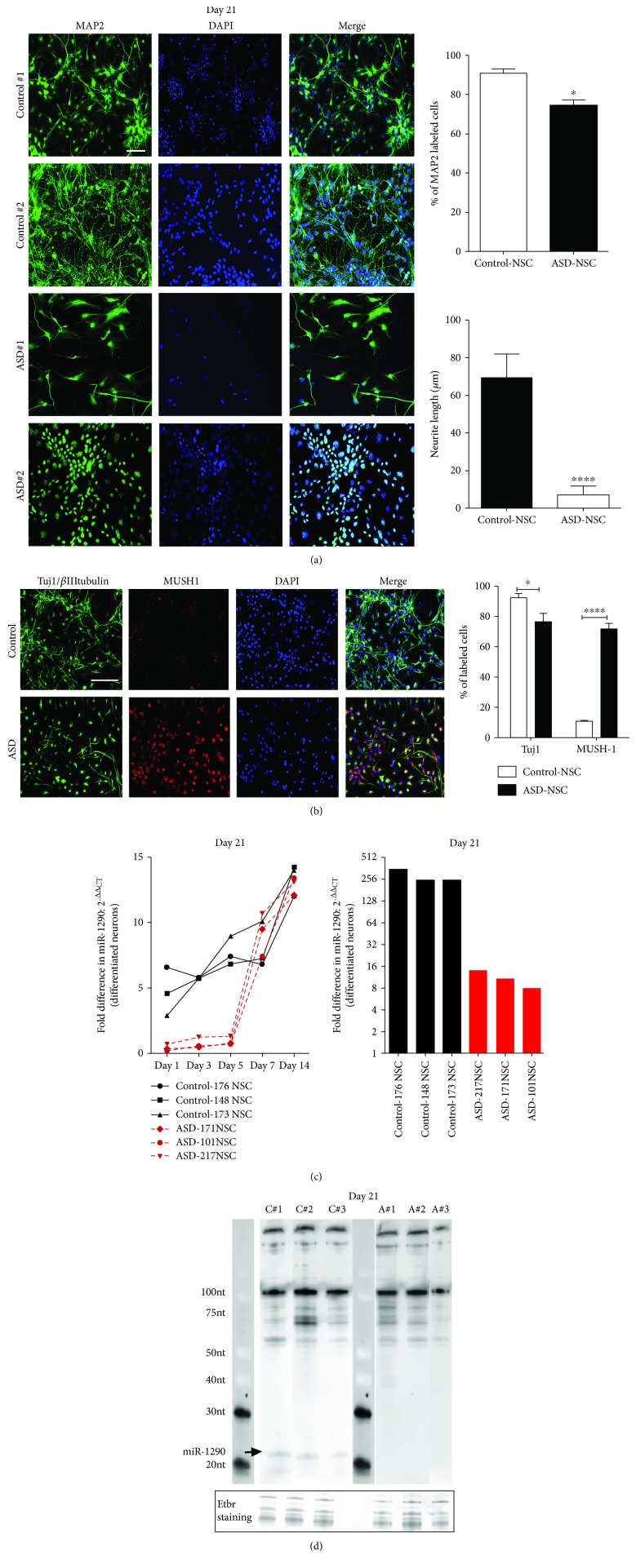
ASD-NSCs display perturbations in neuronal phenotype during early days of differentiation. (a) Differentiated neurons from ASD-NSCs display shorter neurites when compared to neurons differentiated from control-NSCs. A strong staining of the cell body is seen in neurons derived from ASD-NSCs. Bar = 20 *μ*m. ^∗^ *P* < 0.05 and ^∗∗∗∗^ *P* < 0.0001, *n* = 3, determined by an unpaired *t*-test. (b) Immunostaining for other differentiation markers such as tuj1 or *β*IIItubulin and stem cell marker Mushashi1 (Mush1) also shows differential staining in neurons derived from ASD-NSCs than control-NSCs. Bar = 20 *μ*m. ^∗^ *P* < 0.01 and ^∗∗∗∗^ *P* < 0.0001, *n* = 3, determined by an unpaired *t*-test. (c) RNA was extracted from days 1, 3, 5, 7, 14, and 21, and qRT-PCR was conducted for miR-1290. A stable increase in expression was seen in control-NSCs during days of differentiation from day 1 to day 14 whereas at day 21 a significant downregulation in miR-1290 expression was observed in ASD-NSCs. An unpaired *t*-test followed by the Holm-Sidak multiple correction post hoc test revealed significant differences in days 1 (^∗^ *P* < 0.01), 3 (^∗∗∗∗^ *P* < 0.0001), and 5 (^∗∗∗^ *P* = 0.0005). Analysis on day 21 expression revealed a significance of ^∗∗^ *P* < 0.01 (*n* = 3), determined by an unpaired *t*-test. Data are represented as the mean ± SEM. (d) Representative northern blot of RNA derived from day 21 differentiated from three individual donors from control- and ASD-NSCs; note that the anti-DIG signals for the mature form (∼21 nt) were only seen in the control cases. A prestained small molecular weight miRNA marker was used to monitor RNA size.

IPSC-derived NSCs were plated in equal densities on day 1 and grown for 5 days in culture followed by immunostaining for progenitor markers such as Nestin, Sox2, and Pax6. An increased number of progenitor cells as stained by Pax6 were found in idiopathic ASD-NSCs than control-NSCs (Figure 1(a), upper right graph). Next, NSCs were differentiated into neurons in differentiation media and harvested at days 7 and 21 for immunostaining. Immunostaining for Nestin and Sox2 revealed no differences at day 7 (Figure 1(b), upper panels and graph); however, at day 21, a strong labeling was still evident in ASD cells (Figure 1(b), lower panels and graph). Intriguingly, staining with MAP2 revealed a drastic change in labeling patterns between control and ASD cell lines (Figure 2(a), upper bar graph). In control-NSCs, MAP2 staining was found clearly along the cell body and neuronal processes whereas in ASD-NSCs the staining was primarily concentrated in the cytoplasm and nucleus followed by a significant decrease in neurite processes in ASD lines (Figure 2(a), lower bar graph). Further, immunostaining with differentiation marker Tuj1 and proliferation marker Mushashi-1 (MUSH-1) confirmed the presence of mitotically active cells in the neuronal culture derived from ASD-NSCs (Figure 2(b), bar graph). Next, we compared control-NSCs and ASD-NSCs for miR-1290 expression during differentiation. In control-NSCs, we saw a steady rise in miR-1290 levels during different days of differentiation (Figure 2(c), left graph), and the highest expression was observed at day 21 with a ~250-fold increase (Figure 2(c), right graph) whereas in ASD-NSCs, the miR-1290 levels went up steadily till day 14 and remained unaltered till day 21, reaching only a ~15-fold increase (Figure 2(c), right graph). Furthermore, northern blot was performed on day 21-differentiated neurons. Results indicate a significant increase in the ~23 nt band corresponding to the mature miR-1290 only in differentiated control-NSCs and absent from neurons differentiated from ASD-NSCs (Figure 2(d)). These data denote that miR-1290 expression is perturbed during neuronal differentiation in NSCs derived from idiopathic ASD cases.

### 3.2. miR-1290 Overexpression or Coculture with Control-NSCs Failed to Rescue Differentiation Deficits in ASD-NSCs

**Figure 3:**
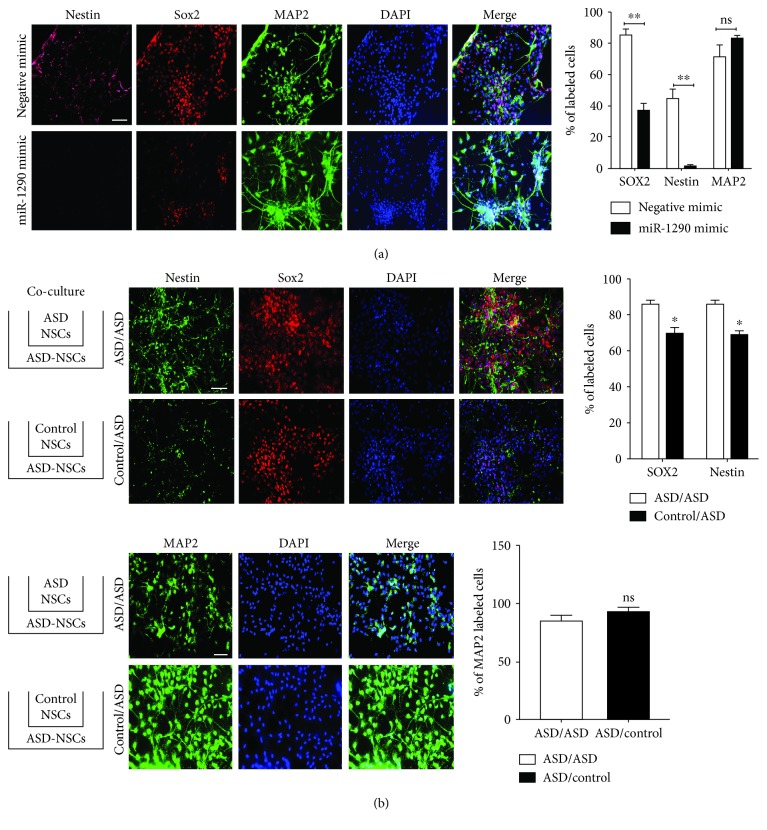
Overexpression of coculture rescued the phenotype (a) Undifferentiated ASD-NSCs were plated and differentiated for 21 days in the presence of miR-1290 and negative mimics followed by immunostaining. Results indicate a significant decrease in progenitor cells but no difference in MAP2-expressing cells was seen. Bar = 20 *μ*m. ^∗∗^ *P* < 0.001 (*n* = 3 donors), determined by an unpaired *t*-test and corrected by the Holm-Sidak multiple correction post hoc test. (b) Coculture with control-NSCs decreased significantly the progenitor cells (Nestin, Sox2) whereas no effect was seen on neuronal MAP2 cells. ^∗^ *P* < 0.01 (*n* = 3 donors), determined by an unpaired t-test and corrected by the Holm-Sidak multiple correction post hoc test. Data are represented as the mean ± SEM.

To test whether miR-1290 overexpression can rescue the differentiation process, ASD-NSCs were transfected with synthetic miR-1290 and negative mimics followed by differentiation for 21 days. While a significant reduction of Nestin and Sox2 positive cells was seen, no increase of MAP2 positive neurons was observed (Figure 3(a)). Since transfection with synthetic mimics failed to rescue differentiation defect in ASD-NSCs, we asked if other additional external factors, present in differentiating control-NSCs but absent from ASD-NSCs, would rescue differentiation. We hypothesized that by supplementing with secretory factors from differentiating control-NSCs, we potentially could rescue these deficits. Control-NSCs were cultured in transwells along with ASD-NSCs, and neuronal differentiation was simultaneously achieved in 21 days. At day 21, ASD-differentiated neurons were fixed and stained for progenitor markers Nestin and Sox2 and neuronal marker MAP2. Results indicated a reduction in Nestin and Sox2-expressing progenitor cells when cocultured with control-NSCs and not ASD-NSCs (Figure 3(b), upper panels); however, no significant increase in expression of MAP2-positive cells was seen (Figure 3(b), lower panels). These data indicate that neither miR-1290 overexpression nor secretory factors alone are sufficient to rescue neuronal differentiation deficits seen in ASD-NSCs.

### 3.3. miR-1290 Packed in Artificial Extracellular Vesicle-Like Particles Rescues Differentiation Deficits in ASD-NSCs

**Figure 4:**
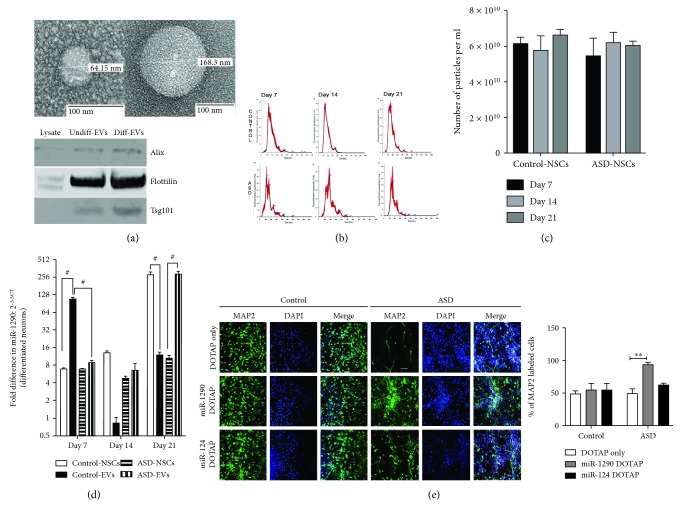
mir-1290 is secreted in extracellular vesicles (EVs). (a) Top: transmission electron microscopy (TEM) of EVs isolated from NSCs shows the presence of different sizes of EVs isolated from control-NSC culture media. Bottom: western blot of EVs from NSC whole cell lysate (lysate) and undifferentiated (undiff) and differentiated (diff) EVs shows the presence of EV markers Alix, Flotillin, and Tsg101 enriched in the EV fraction. (b, c) Nanoparticle tracking analysis (NTA, nanosight) revealed no significant differences in the concentration of the particles between control- and ASD-NSC-derived EVs (*n* = 3 donors). (d) RT-PCR data indicates that miR-1290 levels are significantly upregulated in EVs isolated from day 21-differentiated ASD-NSC when compared to the EVs isolated from control-NSCs. # represents significance. ^∗∗∗∗^ *P* < 0.0001 (days 7 and 14) and ^∗∗^ *P* < 0.001 (day 21) (*n* = 3 donors), determined by an unpaired *t*-test and corrected by the Holm-Sidak multiple correction post hoc test. (e) Undifferentiated ASD-NSCs were plated and differentiated for 21 days in the presence of miR-1290, miR-124, and negative mimics complexed with DOTAP followed by immunostaining with neuronal marker MAP2. Results indicate a significant increase in MAP2-expressing cells only in ASD cultures treated with DOTAP-miR-1290. Bar = 20 *μ*m. ^∗∗^ *P* < 0.001 (*n* = 3 donors), determined by two-way ANOVA followed by Sidak's multiple correction test. Data are represented as the mean ± SEM.

We wondered whether synthetic miRNAs remained functionally active in long-term cultures. A recent discovery of miRNA delivery through extracellular vesicles (EVs) showed that EV-associated miRNAs are more stable and resistant to RNAses than free circulating miRNAs [10, 11]. EVs were isolated from control and ASD-NSC media at various time points during differentiation. Transmission electron microscopy (TEM) and western blotting for EV markers such as Alix, TSG101, and Flotillin revealed the presence of EVs in the media supernatants from undifferentiated (undiff) and 21 days differentiated control-NSCs (Figure 4(a), TEM and western blot). Nanoparticle tracking analysis (NTA, Nanosight) revealed no significant differences in the EV numbers at different days of differentiation either from control or ASD-NSCs (Figures 4(b) and 4(c)). RNA was prepared from both EVs and cells at various time points. As seen in Figure 4(d), while control-NSCs significantly secreted miR-1290 in EVs at day 7, (~250-fold), a similar level of miR-1290 secretion in EVs was observed in ASD-NSCs at day 21. We then examined the possibility to rescue the phenotype by introducing miR-1290 by EV-like particles. We used an artificial liposomal delivery system, DOTAP (N-[1-(2,3-dioleoyloxy)propyl]-N,N,N-trimethylammonium methyl-sulfate) as previously described by our group [12]. After, 48 h post-DOTAP treatment, the ASD-NSCs were cocultured in the presence of control-NSCs. Differentiation was carried out for 21 days after which cells were harvested and stained for Sox2 and MAP2 antibodies. Apart from miR-1290, we also used an evolutionary conserved neuron-specific miRNA, miR-124, as an additional control [13]. As revealed in Figure 4(e), a significant reduction in Sox2-positive cells and a concurrent increase in MAP2-positive cells in DOTAP:miR1290-treated cells were observed when compared to DOTAP controls or DOTAP:miR124 treatments. These experiments clearly suggest that introduction of miR-1290 by EV-like particles in the presence of secretory factors from control-NSCs can significantly aid the promotion of neuronal differentiation in ASD-NSCs.

### 3.4. miR-1290 Targets Several Crucial Genes in Brain Development and ASD

**Figure 5:**
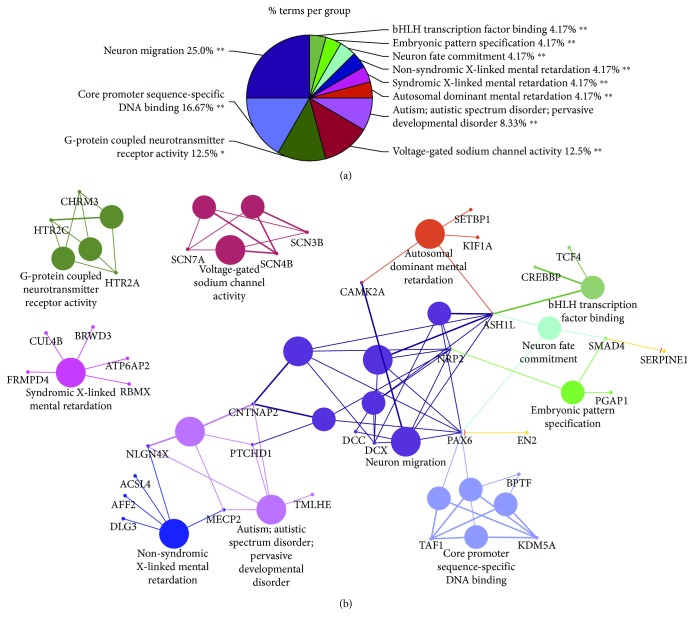
Enriched GO terms (in “cellular process” category) corresponding to 67 target genes associated with ASD. (a) Pie chart representing broad functional groups. The size of each category within a pie chart represents the percent of included terms. All enriched GO terms are statistically significant (*P* value < 0.01). Single and double asterisks indicate significant enriched GO terms at the group with *P* value = 0.01 and the group with *P* value < 0.01, respectively. (b) Networks of functional groups (shown in 5A) with matching color coding. Nodes with kappa score ≥ 0.6, where only the label of the most significant term per functional group is shown. Color-filled large circles represent GO terms, and the circle size represents their enrichment significance. Tiny circles with red text label represent genes associated with one or multiple GO terms. All enriched GO terms shown in the network are statistically significant with a Benjamini-Hochberg corrected *P* value of <0.01. These results are also shown in Table S2 under column “F.”

**Figure 6:**
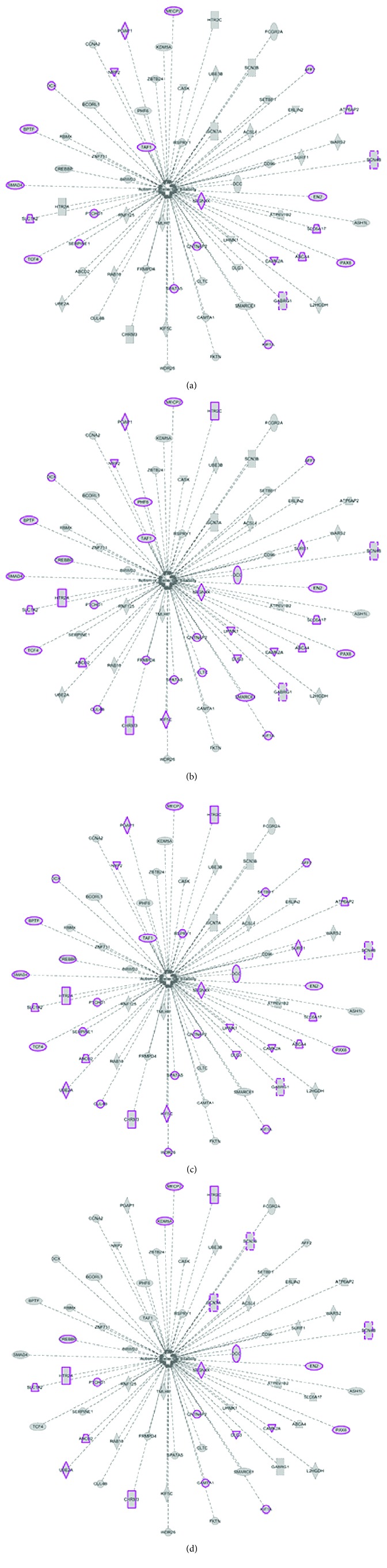
Genes involved in different developmental processes are highlighted over the 67 target genes associated with ASD, using an Ingenuity Pathway Analysis (IPA) tool. (a) Embryonic development. (b) Nervous system development and function. (c) Organismal development. (d) Behavior.

Finally, an important question that needs to be answered is the relationship between miR-1290 expression and ASD. To further understand the relationship between ASD and miR-1290, we performed a global gene target search. We examined the 3′-UTRs of all human mRNA transcripts for complementarity to the miR-1290 seed region. mir-1290 target genes were identified from multiple sources including miRTarBase, mirDB, IPA, and Targetscan to create a combined unique list of 4,644 genes. These gene targets were then examined for enrichment in Gene Ontology categories. Bioinformatic analysis revealed that many of the target genes were present in functional categories related to brain developmental processes such as neuron migration, differentiation, neural fate commitment, and neurogenesis. Of these, a reviewed set of 1,741 genes was further selected and mapped to a curated set of 603 ASD (http://asd.princeton.edu) genes to obtain the final set of 67 mir-1290-targeted ASD genes. Intriguingly, a total of verified 67 genes that also contain exact seed sequence complementarity to miR-1290 are involved directly in key aspects of development and ASD (Table S1, Figure 5). Further, the analysis of the pathways showed clearly that the genes are involved directly in autism and ASD (Table S2). Furthermore, results from IPA analysis suggest that dysregulation of miR-1290 could be a major contributing upstream factor of many significant cellular processes such as embryonic development, nervous system development, organismal development, and behavior that are associated with ASD (Figure 6).

## 4. Discussion

Approximately 46% of human-specific miRNAs which are evolutionarily young or poorly conserved across species play a critical role in the emergence of human diseases [3]. Building on our previous study where we elucidated the role of a young miRNA miR-1290 in neural stem cell proliferation and neuronal differentiation processes [4], our current work further expands its role in delineating its pathological relevance in ASD. We showed that knockdown of miR-1290 led to perturbations in cell cycle processes by targeting crucial cell cycle genes ultimately leading to untimely cell death. A study on human mesenchymal stem cells also reported a significant increase in miR-1290 expression during neuronal differentiation [14], supporting our hypothesis that miR-1290 plays a significant role in differentiation. Intriguingly, recent studies revealed global alterations in cell proliferation, neuronal differentiation, and synaptic assembly in NPCs (neural progenitor cells) differentiated from ASD patient-derived iPSCs [5, 6, 8]. In order to investigate a possible role for miR-1290 in ASD, we utilized neural stem cells (NSCs) from a clinically well-defined population of idiopathic ASD-specific patients and unaffected subjects [9]. We observed not only an abnormal increase in NSC proliferation in cells derived from ASD patients (Figure 1) but also a concurrent decrease in MAP2, and miR-1290 expression was revealed (Figure 2). Interestingly, in our previous publication, we reported a highest fold change in differentiated neurons to be around ~14-fold. The discrepancy in the fold changes could be due to the fact that we are using IPSCs and not fetal-derived neural progenitors to differentiate. Northern blot further confirmed the presence of mature miR-1290 only in differentiated ASD-NSCs. The presence of more than 80% proliferating cells in the differentiated cultures could be debated as possible technical artifact or clonal artifacts or different culture conditions. Certainly, more work needs to be done on several different clones to see if this is a valid observation; nevertheless, a result from northern blot analysis clearly indicating the absence of mature form in day21 cultures suggests that there is, in fact, a valid differentiation problem in all the three ASD clones. Interestingly, the primary transcripts are present in both control and ASD lines indicating a dysregulation in miRNA processing in day 21-differentiated immature neurons, obtained from ASD-NSCs. A recent study on IPSC-derived NSCs from a selected cohort of macrocephalic ASD subjects revealed that ASD-associated temporal dysregulation within a specific neurodevelopmental gene module caused marked changes in the maturational sequence of cortical neuron development, including morphological growth acceleration and heterochronic initiation of the neuronal program [15], indicating an accelerated early differentiation that clearly contradicts our results. This discrepancy could be because the IPSC clones used in our study were isolated from patients characterized under “idiopathic ASD” that do not display macrocephaly. However, future works on selected groups of phenotypes are essential to delineate the function of miR-1290 in ASD. Hence, it is important to maintain caution when extrapolating our findings to other findings in the field as patient variability, clonal differences, or experimental conditions (e.g., neuronal differentiation timeline) can largely affect the outcome. Hence, the need for more personalized therapy for treatment of disorders such as ASD is undermined. Although this study utilizes a small cohort size, 3 individuals per group and NSCs derived from one IPSC clone per individual, the robustness of the expression data as indicated both by RT-PCR and northern blot confirms that the expression of miR-1290 is indeed perturbed in ASD cell lines. A recent study comparing the robustness of transcriptomic data sets obtained from IPSC disease model studies which published either single or multiple IPSC clones indicated that widespread use of more than one clone per individual in combination with current analytical practices is indeed detrimental to the robustness of the findings [16]. Previously, we used NPCs isolated directly from human fetal brains, and here, we used NSCs derived from iPSCs. Nevertheless, both these methods indicate that neuronal differentiation in control cell lines increased miR-1290 expression indicating that miR-1290 might be playing a central role during neuronal differentiation. Next, we tested whether overexpressing miR-1290 or coculture with control-NSCs could rescue differentiation deficits in ASD-NSCs. Intriguingly, neither of the approaches affected neuronal differentiation although a significant decrease in progenitor cells was seen. We previously published that miRNAs are secreted in EVs [12, 17]. We tested whether miR-1290 is released in EVs, and indeed, we see a disparity in miR-1290 release between control and ASD-NSCs during differentiation. To rescue the levels of miR-1290, we utilized artificial EV-like liposomal (DOTAP) particles in a coculture set-up. We and others have shown that this kind of delivery is successful in neurons for long-term delivery of nucleotides such as miRNAs, which can be delivered in their unmodified form into neurons by endocytosis and released into the cytoplasm thus mimicking EV delivery [12, 18, 19]. Indeed, miR-1290 when delivered by DOTAP and when cocultured with control-NSCs clearly restored miR-1290 levels in ASD-NSCs. It is interesting that miR-124 which is discussed as a common neural inducer was unable to rescue differentiation in our model. The discrepancy could be due to the fact that other key transcription factors were not transfected along with miR-124 as published previously [20]. Further, most of the studies using miR-124 were conducted either in cellular models derived from mouse [21, 22] or in humans derived from stem cells originating from different cellular origins such as mesenchymal stem cells [23]. Finally, we performed a comprehensive bioinformatic analysis to reveal gene targets that can help us identify the biological function and the relationship of miR-1290 to ASD. We identified 67 gene targets that not only are crucial in brain development and related pathways but also have been reported to be altered in ASD. For example, miR-1290 directly can target genes such as MECP2, NLG3, DCX, DLG3, and CNTNAP2, all implicated with ASD [24–28]. However, it is intriguing to note that previous studies from various groups showed no significant differences in the differentiated neuron number obtained from ASD patients [5, 6, 29, 30]. This discrepancy in our study can be due to the age of the differentiated neurons. For example, the study from Marchetto et al. [6] used cultures that were 6-7week old and Mariani et al. [5] worked on differentiated neurons at day 31. In this study, we monitored neurons that are still undergoing differentiation (day 7 and day 21) and therefore should be considered immature neurons. Nevertheless, it is important to note that perturbations in miR-1290 expression early on during differentiation could affect its target gene expression affecting the function of the mature neurons, shown by synaptic deficits and aberrant electrophysiology as shown in previous studies. It will be interesting to test whether reinstatement of miR-1290 levels through EV-like particles would rescue such deficits in the mature neurons.

## 5. Conclusions

Based on our earlier work demonstrating its role in cell cycle regulation, proliferation, and differentiation [4] and the current work showing its ability to be secreted into EVs and to target potential ASD genes, we conclude that miR-1290 could play a key role during brain development. In summary, this study highlights the significance of using human ASD patient-derived reprogrammed iPSCs and NSCs to study functional roles of higher-specific miRNAs such as miR-1290 in human brain disorders.

## Figures and Tables

**Table 1:** 

Antibody name	Dilution, method	Company, catalog number
Map2	1 : 2000, ICC	Abcam, ab5392
Nestin	1 : 1000, ICC	Millipore, MAB5326
Sox2	1 : 1000, ICC	Millipore, MAB5603
NeuN	1 : 500, ICC	Millipore, MAB377
BetaIIItubulin/TUJ	1: 1000, ICC	Millipore, AB9354
Pax6	1 : 500, ICC	Covance, PRB-278P
Flotillin	1 : 1000, WB	Abcam, ab41927
TSG101	1 : 500, WB	SBI, EXOAB-TSG101-1
Alix	1 : 500,WB	BioLegend, 634501

## Data Availability

No data were used to support this study.

## Supplementary Materials

Supplementary Materials Table S1: a list of reviewed set of 67 miR-1290 target genes that were obtained by mapping to a curated set of ASD genes. Table S2: the table shows all enriched GO terms shown in the network which are statistically significant with a Benjamini-Hochberg-corrected *P* value of <0.01.
