# Supplementary material for: Downregulation of an Evolutionary Young miR-1290 in an iPSC-Derived Neural Stem Cell Model of Autism Spectrum Disorder
Source: Stem Cells Int. 2019 May 2;2019:8710180. doi: 10.1155/2019/8710180 (PMC6525818; doi:10.1155/2019/8710180)
Supplement: Supplementary Materials — Table S1: a list of reviewed set of 67 miR-1290 target genes that were obtained by mapping to a curated set of ASD genes. Table S2: the table shows all enriched GO terms shown in the network which are statistically significant with a Benjamini-Hochberg-corrected P value of <0.01. [file 8710180.f1.pdf]

Table S1. A list of reviewed set of 67 miR-1290 target genes that were obtained by mapping to a curated set of ASD genes.

© 2000-2018 QIAGEN. All rights reserved.

ID	Symbol	Entrez Gene Name	Location	Type(s)
ABCA4	ABCA4	ATP binding cassette subfamily A member 4	Plasma Membrane	transporter
ABCD2	ABCD2	ATP binding cassette subfamily D member 2	Cytoplasm	transporter
ACSL4	ACSL4	acyl-CoA synthetase long chain family member 4	Cytoplasm	enzyme
AFF2	AFF2	AF4/FMR2 family member 2	Nucleus	other
ASH1L	ASH1L	ASH1 like histone lysine methyltransferase	Nucleus	transcription regulator
ATP6AP	ATP6AP2	ATPase H+ transporting accessory protein 2	Cytoplasm	transporter
ATP6V1	ATP6V1B	ATPase H+ transporting V1 subunit B2	Cytoplasm	transporter
BCORL1	BCORL1	BCL6 corepressor like 1	Nucleus	transcription regulator
BPTF	BPTF	bromodomain PHD finger transcription factor	Nucleus	transcription regulator
BRWD3	BRWD3	bromodomain and WD repeat domain containing 3	Other	other
CAMK2A	CAMK2A	calcium/calmodulin dependent protein kinase II alpha	Cytoplasm	kinase
CAMTA1	CAMTA1	calmodulin binding transcription activator 1	Other	other
CASK	CASK	calcium/calmodulin dependent serine protein kinase	Plasma Membrane	kinase
CCNA2	CCNA2	cyclin A2	Nucleus	other
CD96	CD96	CD96 molecule	Plasma Membrane	other
CHRM3	CHRM3	cholinergic receptor muscarinic 3	Plasma Membrane	G-protein coupled receptor
CLTC	CLTC	clathrin heavy chain	Plasma Membrane	other
CNTNAP	CNTNAP	contactin associated protein like 2	Plasma Membrane	other
CREBBP	CREBBP	CREB binding protein	Nucleus	transcription regulator
CUL4B	CUL4B	cullin 4B	Nucleus	other
DCC	DCC	DCC netrin 1 receptor	Plasma Membrane	transmembrane receptor
DCX	DCX	doublecortin	Cytoplasm	other
DLG3	DLG3	discs large MAGUK scaffold protein 3	Plasma Membrane	kinase
EN2	EN2	engrailed homeobox 2	Nucleus	transcription regulator
ERLIN2	ERLIN2	ER lipid raft associated 2	Plasma Membrane	kinase
FCGR2A	FCGR2A	Fc fragment of IgG receptor IIa	Plasma Membrane	transmembrane receptor
FKTN	FKTN	fukutin	Extracellular Space	other
FRMPD4	FRMPD4	FERM and PDZ domain containing 4	Other	other
GABRG1	GABRG1	gamma-aminobutyric acid type A receptor gamma1 su	Plasma Membrane	ion channel
HTR2A	HTR2A	5-hydroxytryptamine receptor 2A	Plasma Membrane	G-protein coupled receptor
HTR2C	HTR2C	5-hydroxytryptamine receptor 2C	Plasma Membrane	G-protein coupled receptor
KDM5A	KDM5A	lysine demethylase 5A	Nucleus	transcription regulator
KIF1A	KIF1A	kinesin family member 1A	Cytoplasm	other
KIF5C	KIF5C	kinesin family member 5C	Cytoplasm	enzyme
L2HGDH	L2HGDH	L-2-hydroxyglutarate dehydrogenase	Cytoplasm	enzyme
MECP2	MECP2	methyl-CpG binding protein 2	Nucleus	transcription regulator
NLGN4X	NLGN4X	neuroligin 4, X-linked	Plasma Membrane	enzyme
NRP2	NRP2	neuropilin 2	Plasma Membrane	kinase
PAX6	PAX6	paired box 6	Nucleus	transcription regulator
PGAP1	PGAP1	post-GPI attachment to proteins 1	Cytoplasm	enzyme
PHF6	PHF6	PHD finger protein 6	Nucleus	transcription regulator
PTCHD1	PTCHD1	patched domain containing 1	Plasma Membrane	other
RAB18	RAB18	RAB18, member RAS oncogene family	Cytoplasm	enzyme
RBMX	RBMX	RNA binding motif protein, X-linked	Nucleus	other
RNF125	RNF125	ring finger protein 125	Cytoplasm	enzyme
RSPRY1	RSPRY1	ring finger and SPRY domain containing 1	Other	other
SCN3B	SCN3B	sodium voltage-gated channel beta subunit 3	Plasma Membrane	ion channel
SCN4B	SCN4B	sodium voltage-gated channel beta subunit 4	Plasma Membrane	ion channel
SCN7A	SCN7A	sodium voltage-gated channel alpha subunit 7	Plasma Membrane	ion channel
SERPINI	SERPINE	serpin family E member 1	Extracellular Space	other
SETBP1	SETBP1	SET binding protein 1	Nucleus	other
SLC1A2	SLC1A2	solute carrier family 1 member 2	Plasma Membrane	transporter
SLC6A1	SLC6A17	solute carrier family 6 member 17	Cytoplasm	transporter
SMAD4	SMAD4	SMAD family member 4	Nucleus	transcription regulator
SMARCE	SMARCE	SWI/SNF related, matrix associated, actin dependent r	Nucleus	transcription regulator
SPATA5	SPATA5	spermatogenesis associated 5	Cytoplasm	other
SURF1	SURF1	SURF1, cytochrome c oxidase assembly factor	Cytoplasm	enzyme
TAF1	TAF1	TATA-box binding protein associated factor 1	Nucleus	transcription regulator
TCF4	TCF4	transcription factor 4	Nucleus	transcription regulator
TMLHE	TMLHE	trimethyllysine hydroxylase, epsilon	Cytoplasm	enzyme
UBE2A	UBE2A	ubiquitin conjugating enzyme E2 A	Cytoplasm	enzyme
UBE3B	UBE3B	ubiquitin protein ligase E3B	Extracellular Space	enzyme
UHMK1	UHMK1	U2AF homology motif kinase 1	Nucleus	kinase
WARS2	WARS2	tryptophanyl tRNA synthetase 2, mitochondrial	Cytoplasm	enzyme
WDR26	WDR26	WD repeat domain 26	Cytoplasm	other
ZBTB24	ZBTB24	zinc finger and BTB domain containing 24	Nucleus	other
ZNF711	ZNF711	zinc finger protein 711	Nucleus	other

Table S2. The table shows all enriched GO terms shown in the network which are statistically significant with a Benjamini-Hochberg corrected p-value of < 0.01.

GO Groups	GOID	GOTerm	Ontology Source	Term PValue	Term PValue Corrected with Benjamini-Hochberg	Group PValue	Group PValue Corrected with Benjamini-Hochberg	GOLevels	% Associated Genes	Nr. Genes	Associated Genes Found
Group00	GO:0043425	bHLH transcription factor binding	GO_MolecularFunction-EBI-UniProt-GOA_10.07.2018_00h00	0.0005	0.0010	0.0005	0.0009	[4]	6.82	3.00	[ASH1L, CREBBP, TCF4]
Group01	GO:0009880	embryonic pattern specification	GO_BiologicalProcess-EBI-UniProt-GOA_10.07.2018_00h00	0.0029	0.0034	0.0029	0.0040	[3, 4, 5]	3.75	3.00	[NRP2, PGAP1, SMAD4]
Group02	GO:0048663	neuron fate commitment	GO_BiologicalProcess-EBI-UniProt-GOA_10.07.2018_00h00	0.0035	0.0037	0.0035	0.0043	[4, 5, 7, 8, 9]	3.53	3.00	[ASH1L, PAX6, SMAD4]
Group03	ds:H00480	Non-syndromic X-linked mental retardation	KEGG-HUMAN-DISEASE_10.07.2018	0.0000	0.0000	0.0000	0.0000	[-1]	19.23	5.00	[ACSL4, AFF2, DLG3, MECP2, NLGN4X]
Group04	ds:H00658	Syndromic X-linked mental retardation	KEGG-HUMAN-DISEASE_10.07.2018	0.0000	0.0000	0.0000	0.0000	[-1]	17.24	5.00	[ATP6AP2, BRWD3, CUL4B, FRMPD4, RBMX]
Group05	ds:H00773	Autosomal dominant mental retardation	KEGG-HUMAN-DISEASE_10.07.2018	0.0000	0.0002	0.0000	0.0001	[-1]	7.69	4.00	[ASH1L, CAMK2A, KIF1A, SETBP1]
Group06	ds:H02111	Autism; Autistic spectrum disorder; Pervasive developmental disorder	KEGG-HUMAN-DISEASE_10.07.2018	0.0000	0.0000	0.0000	0.0000	[-1]	45.45	5.00	[CNTNAP2, MECP2, NLGN4X, PTCHD1, TMLHE]
Group07	GO:0005248	voltage-gated sodium channel activity	GO_MolecularFunction-EBI-UniProt-GOA_10.07.2018_00h00	0.0002	0.0005	0.0036	0.0040	[7, 8, 9]	9.68	3.00	[SCN3B, SCN4B, SCN7A]
Group08	GO:0099528	G-protein coupled neurotransmitter receptor activity	GO_MolecularFunction-EBI-UniProt-GOA_10.07.2018_00h00	0.0005	0.0010	0.0135	0.0135	[4, 5]	6.82	3.00	[CHRM3, HTR2A, HTR2C]
Group09	GO:0001046	core promoter sequence-specific DNA binding	GO_MolecularFunction-EBI-UniProt-GOA_10.07.2018_00h00	0.0007	0.0010	0.0015	0.0023	[7, 8]	6.25	3.00	[KDM5A, PAX6, TAF1]
Group10	GO:0001764	neuron migration	GO_BiologicalProcess-EBI-UniProt-GOA_10.07.2018_00h00	0.0001	0.0003	0.0002	0.0004	[4, 5, 6, 7, 8]	3.02	6.00	[ASH1L, CAMK2A, DCC, DCX, NRP2, PAX6]
